# STATE-LEVEL OPPORTUNITIES TO ADDRESS BEHAVIORAL HEALTH NEEDS OF OLDER ADULTS: A NEW CENTER OF EXCELLENCE FOR OREGON

**DOI:** 10.1093/geroni/igae098.3894

**Published:** 2024-12-31

**Authors:** Walter Dawson, Allyson Stodola, Karen Cellarius, Dani Himes, Lindsey Smith, Leah Brandis, Nirmala Dhar, Paula Carder

**Affiliations:** Oregon Health & Science University, Portland, Oregon, United States; Portland State University, Portland, Oregon, United States; Portland State University, Portland, Oregon, United States; Portland State University, Portland, Oregon, United States; Oregon Health & Science University, Portland, Oregon, United States; OHSU, Portland, Oregon, United States; Oregon Health Authority, Portland, Oregon, United States; OHSU-PSU School of Public Health, Portland, Oregon, United States

## Abstract

Serious mental illness and substance use disorders among older adults frequently intersect with neurocognitive changes and complex physical health issues. At the same time, access to behavioral health services is often poor for many older adults. Multiple underserved populations such as older adults from communities of color and individuals living in rural/remote communities experience substantial barriers to access and use of behavioral health services. There is a need for enhanced program and policy actions to ensure access to behavioral health for these populations of older adults. This session describes the state of behavioral health and aging services for older adults in Oregon and efforts to address local and statewide needs. This includes the impetus for establishing the Oregon Center of Excellence for Behavioral Health and Aging (OCEBHA), the goals of OCEBHA, and the long-term vision of the Center to address behavioral health disparities.

